# Modeling of variability and uncertainty in human health risk assessment

**DOI:** 10.1016/j.mex.2017.01.005

**Published:** 2017-01-31

**Authors:** Palash Dutta

**Affiliations:** Dept. of Mathematics, Dibrugarh University, Dibrugarh, 786004, India

**Keywords:** Variability and uncertainty, Generalized fuzzy numbers, Interval valued fuzzy numbers, Risk assessment

## Abstract

Health risk assessments have been carried out worldwide to examine potential health risk due to exposure to toxic contaminants in various environments. In risk assessment, it is most important to know the nature of all available information, data or model parameters. It is observed that available information/data are tainted with uncertainty and variability in the same time, i.e., uncertainty and variability co-exist. In such situation it is important to devise method for processing both uncertainty and variability into same framework and which is an open issue. In this regards, this paper presents an algorithm to combined approach to propagate variability and uncertainty in the same framework. The differences and advantages of this algorithm over the existing methods are presented below:

• The representation of uncertain model parameters are probabilistic together with generalized fuzzy numbers and normal interval valued fuzzy numbers.
• The results obtained are then interpreted in terms of p-box and fuzzy numbers.
• The advantage of this approach over the existing methods is that this approach gives an accurate resultant fuzzy number which is of trapezoidal type generalized fuzzy number that is different from the existing methods.

## Graphical abstract

**Figure:**
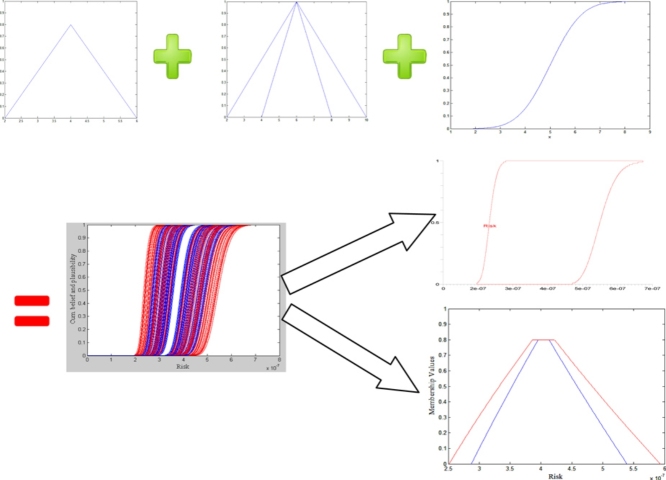

## Uncertainty modeling approaches

Uncertainty is an integral and unavoidable part of risk assessment. Based on the nature and availability of data variability and uncertainty can be modeled using probability theory and fuzzy set theory.

## Probability theory

Probability theory frequently used in variability analysis. If parameters used in prescribed models are random in nature and followed well define distribution, then probabilistic methods are most suitable and well accepted approach for risk assessment.

This approach can describe variability arising from stochastic disturbances, variability conditions, and risk considerations. Variability is characterized by the probability associated with events. The probability of an event can be interpreted in terms of frequency of occurrence which can be defined as the ratio of the number of favorable events to the total number of events. In this approach, the uncertainties associated with model inputs are described by probability distributions, and the objective is to estimate the output probability distributions.

A random variable is a variable in a study in which subjects are randomly selected. Let *X* be a discrete random variable.

A probability mass function is a function such that

$$\left(i\right)\overset{}{\underset{}{\;f\left(x_{i}\right)\geq 0,\;\left(ii\right)\overset{n}{\underset{i=1}{\;\sum }}}}f\left(x_{i}\right)=1\;\;\left(iii\right)\;f\left(x_{i}\right)=p\left(x=x_{i}\right)$$

The cumulative distribution function of a discrete random variable X, denoted as F(x) is

$$F(x)=P(X\leq x)=\sum_{x^{}\leq x_{i}}f{(x}_{i})$$

Let *X* be a continuous random variable. A probability density function of *X* is a non-negative function *f*, which satisfies

$$P(X\in B)=\int_{B}f(x)dx$$

for every subset B of the real line.

As *X* must assume some value, f must satisfy

$$P(X\in (-\infty ,\infty )=\int_{-\infty }^{\infty }f(x)dx=1$$

This means the entire area under the graph of the PDF must be equal to unit.

In particular, the probability that the value of *X* falls within an interval [a, b] is

$$p(a\leq X\leq b)=\int_{a}^{b}f(x)dx$$

The CDF of a continuous random variable *X* is

$$F(x)=P(X\leq x)=\int_{-\infty }^{x}f(x)dx$$

## Fuzzy set theory

Environmental/human health risk assessment is an important aid in any decision-making process in order to minimize the effects of human activities on the environment. Unfortunately, in general environmental data tends to be vague and imprecise, so uncertainty is associated with any study related with these kinds of data. Fuzzy set theory provides a way to deal with the imprecisely defined variables; defined relationships between variables based on expert human knowledge and use them to compute results. In this section, some necessary backgrounds and notions [1], [2] of fuzzy set theory that will be required in the sequel are reviewed.

**Definition:** Let *X* be a universal set. Then the fuzzy subset *A* of *X* is defined by its membership function

$$\mu_{A}:X\to [0,1]$$

Which assign a real number *μ*_A_(x)in the interval [0,1], to each element *x ∈ A*, where the value of *μ_A_(x)* at *x* shows the grade of membership of *x* in *A.*

**Definition:** Given a fuzzy set *A* in *X* and any real number **α** ∈ [0,1]. Then the **α** −cut or **α** −level or cut worthy set of *A*, denoted by ***^α^*** *A* is the crisp set

$${}^{\alpha }A=\left\{x\in X:\mu_{A}(x)\geq \alpha \right\}$$

**Definition:** The support of a fuzzy set *A* defined on *X* is a crisp set defined as

$$S\mathrm{up}p\;\;\left(A\right)\{x\in X:\mu_{A}(x)>0\}$$

**Definition:** The height of a fuzzy set *A*, denoted by *h*(*A*) is the largest membership grade obtain by any element in the set and it is denoted as

$$h(A)=\underset{x\in X}{\sup}\mu_{A}(x)$$

**Definition:** An interval valued fuzzy set *A* defined in the universe of discourse X is represented by

$$A=\left\{\left(x,\;\left[\mu_{A}^{L}(x),\;\mu_{A}^{U}(x)\right]\;\right):\;x\in X)\right\}$$

Where $0\leq \mu_{A}^{L}(x)\leq \mu_{A}^{U}(x)\leq 1$ and the membership grade ${\bar{\mu }}_{A}(x)$of elements of *x* to the interval valued fuzzy set *A* is represented by an interval $\left[\mu_{A}^{L}(x),\;\mu_{A}^{U}(x)\right]$$\left(i.e.,\;{\bar{\mu }}_{A}(x)=[\mu_{A}^{L}(x),\;\mu_{A}^{U}(x)]\right)\text{.}$

An interval valued fuzzy number (IVFN) is called normal IVFN if heights of both UMF and LMF are exactly equal to 1, it is called generalized IVFN if height of the LMF is less than 1 and it is called completely generalized IVFN if heights of both UMF and LMF are less than 1.

• *A* is normal
• *A* is defined in a closed bounded interval
• *A* is convex set

Then *A* is called an interval valued fuzzy number.

**Definition:** α−Cut of IVFN [2]:

Let A be a continuous and convex IVFN with LMF $\mu_{A}^{L}$(or$\underline{A}$) and UMF $\mu_{A}^{U}$(or$\bar{A}$). Let α−cut of LMF ($\underline{A}$) be A_α=[Lxα¯,≤Rxα_] and of UMF ($\bar{A}$) be A¯α=[Lxα¯,Rxα¯]. Then α−cut of IVFN A can be evaluated by the following formula:

Aα={([Lxα̲,Rxα̲],[Lxα¯,Rxα¯]),  α≤h(A¯)  &  α≤h(A̲). (φ,[Lxα¯,Rxα¯]),α≤h(A¯)  &  α>h(A̲).  (φ,φ),α>h(A¯)  

where$\forall \alpha :L_{\bar{x_{\alpha }}}\leq L_{\underline{x_{\alpha }}}\leq R_{\underline{x_{\alpha }}}\leq R_{\bar{x_{\alpha }}}$,$h(\underline{A})$ is the height of LMF, $h(\bar{A})$ is the height of UMF and *φ* is an empty set.

## Approach to propagate variability and uncertainty

In uncertainty modeling in terms of fuzzy set theory it is observed that representation of uncertain parameters is Type-I fuzzy set in which it is considered that membership functions precisely assign a point value from [0,1].

However, in some situation it is not always possible for a membership function of the type *μ : X*  → [0, 1] to precisely assign one point from [0,1] so it is more realistic to assign interval value. So interval valued fuzzy numbers come into picture.

Here, probability distribution, generalized fuzzy numbers and normal IVFNs have been combined.

Consider an arbitrary mathematical model

$$M=(P_{i},G_{k},F_{l})$$

where *i* = 1, 2, 3, ..., *m* ; *k* = 1, 2, 3, ..., *s* & *l* = 1, 2, 3, ..., *n*

which is a function of parameters. Suppose *P_i_’s* are *m* parameters presented by probabilistic distributions; *G_k_’s* as are *s* parameters presented by generalized fuzzy numbers with heights *w_s_* and *F_l_* are *n* parameters presented by normal interval valued fuzzy numbers (IVFNs).

## The approach is explained below

**Step 1:** Initially, consider all generalized fuzzy numbers *G_k_* with heights *w_s_* as well as upper membership functions *F^u^_1_*, *F^u^_2_*,.,*F^u^_n_* of normal interval valued fuzzy numbers (IVFNs). As generalized fuzzy numbers and normal fuzzy numbers have different heights, so to deal with the model, we consider $\alpha =[0:\frac{w}{10}:\;\;w]$where *w* = min(*w_s_*, 1).

**Step 2:** Generate *5000* numbers of uniformly distributed random numbers from [0,1] and perform Monte Carlo simulation to obtain *m* numbers of cumulative distribution functions (CDFs) from the *m* input random numbers.

**Step 3:** Calculate α-cut for each fuzzy number (α can be taken stepwise from 0 to *w*). Then *s* *+* *n* numbers of closed intervals (as α-cut gives closed intervals) will be obtained.

**Step 4:** Assign all *m* numbers of CDFs and all combination of initial and end points of the *n* + *s* intervals in the model *M* and which will produce 2^*s*+*n*^ of CDFs. Evaluating infimum (minimum) and supremum (maximum) of the model *M* will give a pair of CDFs (i.e., one lower probability distribution and one is upper probability distribution).

**Step 5:** Consider another α level (say, for next α value 0.08 if *w* *=* *0.8*) to calculate α-cut of each fuzzy numbers and repeat step- 3 to step- 4. The process will be terminated after execution for the value α = *w*.

Then, this will produce a family of CDFs.

**Step 6:** Next, consider all generalized fuzzy numbers *G_k_* as well as lower membership functions *F^l^_1_*, *F^l^_2_*,.,*F^l^_n_* of normal interval valued fuzzy numbers *F_l_* respectively. Here also heights of normal and generalized fuzzy numbers are different, we consider that α = [0, *w*] where *w* = min(*w_s_*, 1).

**Step 7:** Repeat step-3 to step-5. In step-6 it should be noted that$\alpha =[0:\frac{w}{10}\;:w]$. Then we shall have another family of CDFs.

Then the final result can be interpreted in two ways. First one is, envelope of all the obtained CDFs or P-box. That is, cumulative distribution functions for some variable lies on or between two monotonic curves, then these curves form a box and called a probability box or P-box for that variable.

Later one is, Membership functions at different fractiles can be generated from these families of cdfs. It will be completely generalized trapezoidal type interval valued fuzzy number. First family of cdfs will produce UMF and later family of cdfs will give LMF with each of height *w* of the resulting completely generalized interval valued fuzzy number generated at different fractiles.

In this approach, 5000 Monte Carlo simulations have been considered. We initially tested with 3000, 4000, 5000 up to 10,000 simulations. Since the results obtained are very similar from 5000 simulations onwards, so, we have decided to consider 5000 Monte Carlo simulations for the rest of the study.

## Hypothetical case study

To demonstrate and make use of the proposed hybrid approach a hypothetical case study for non-cancer risk assessment is carried out here. As due to the discharge of produce water into the sea a lot of organic and inorganic pollutants (however, in this case study we consider only the heavy metal arsenic (As) because of its toxicity and high concentration in produced water.) release into the water and which are harmful to the aquatic organism. Therefore human being may be affected by ingestion of such contaminated aquatic organism. An evaluation is necessary to determine the possible impact such substances may have on human health and ecology. For this purpose, risk assessment is performed to quantify the potential detriment to human and evaluate the effectiveness of proposed remediation measures.

The general form of a comprehensive food chain risk assessment model as provided by EPA U.S. [3] is follows

(1) $$CDI=\frac{C_{f}\times FIR\times FR\times EF\times ED\times CF}{BW\times AT}$$

Where *CDI* = Chronic daily intake (mg/kg-day), *FIR* = fish ingestion rate (g/day), *FR* = fraction of fish from contaminated source, *EF* = exposure frequency (day/year), *ED* = exposure duration (years), *CF* = conversion factor (=10^−9^), *BW* = body weight (kg), *AT* = averaging time (days) and *C_f_* = chemical concentration of fish tissue (mg/kg). The chemical concentration in fish tissue (*C_f_*) can be computed as

(2) $$C_{f}=PEC\times BCF$$

Where *PEC* = predicted environmental concentration (mg/l) and *BCF* is the chemical bioaccumulation factor in fish (l/kg).

The non-cancer risk model for fish ingestion is expressed as:

(3) $$Risk_{non-cancer}=\frac{CDI}{Rfd}$$

Where, *Rfd* is the reference dose.

In this study, representation of the parameters predicted environmental concentration (*PEC*), chemical bioaccumulation factors (*BCF*) are considered to be fuzzy number while fish ingestion rate (*FIR*) is taken as normal probability distribution and other parameters are taken to be constant. Values of the parameters for the calculation of non-cancer risk are given in the Table 1.

**Fig. 1:**
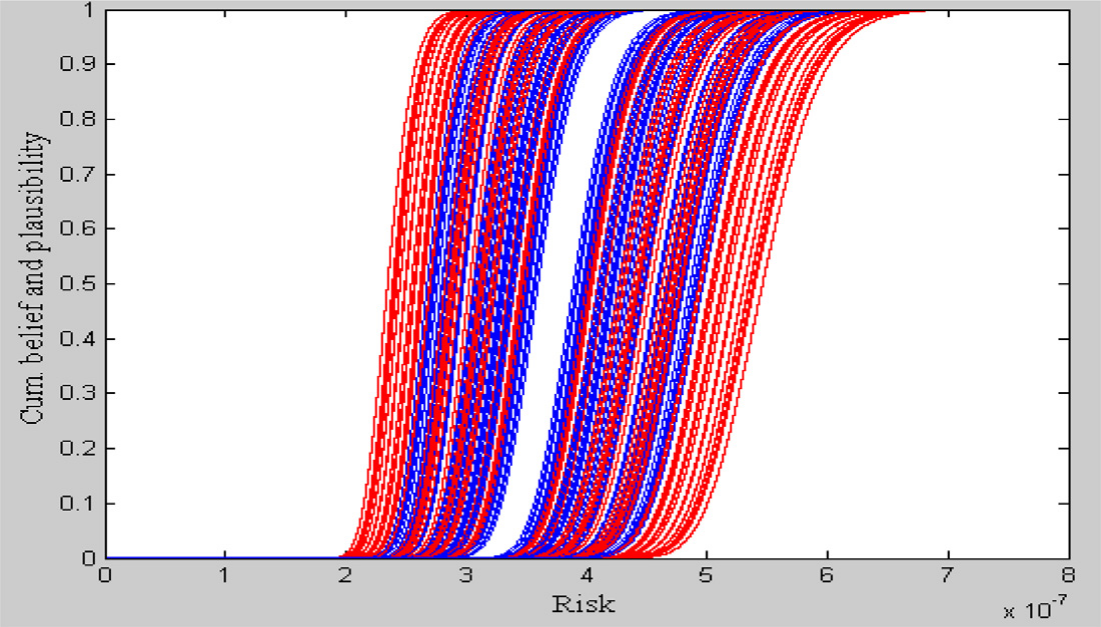
Cumulative distribution functions of non cancer risk for different α-values.

The result of the non-cancer human health risk assessment is performed using our proposed hybrid approach and which is depicted in the following Fig. 1.

The result of the risk assessment is obtained in the form of family of Cdfs (basically two family of cdfs, one in red colored and another in blue colored) at different α-values. Red colored Cdfs are obtained for UMF and blue colored Cdfs are obtained for LMF of the uncertain input parameter *BCF*.

**Fig. 2:**
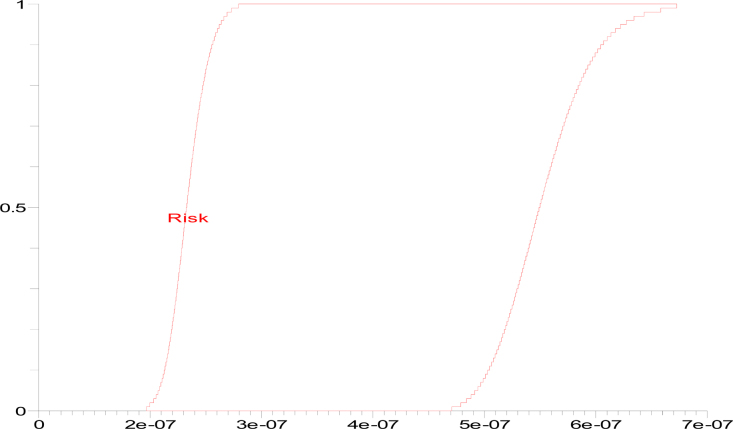
p-box of the CDFs obtained for UMF.

Then, p-box of the CDFs obtained for UMF is given in Fig. 2 whose range is [1.96688e-07, 6.72691e-07], mean is [0.00000023, 0.00000056] and variance is [0,3.36780952e-14].

**Fig. 3:**
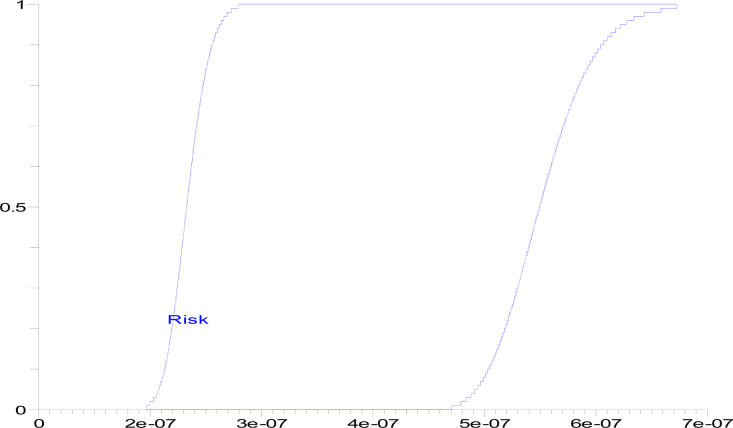
p-box of the CDFs obtained for LMF.

Similarly, p-box of the CDFs obtained for LMF is depicted in Fig. 3 and whose range is [2.24787e-07,6.11537e-07], mean is [0.000000266,0.000000503] & variance is[0,1.996526479e-14].

However, since an envelope is the boundary of all the CDFs and hence resultant envelope/p-box will be p-box obtained for UMFs.

On the other hand, from these cdfs in Fig. 1, risk at different fractiles [1], [4] can be calculated and which are obtained in the form of completely generalized interval valued fuzzy number with each of height 0.8. It is because any arithmetic operations between generalized fuzzy numbers and normal fuzzy numbers produces generalized fuzzy number.

**Fig. 4:**
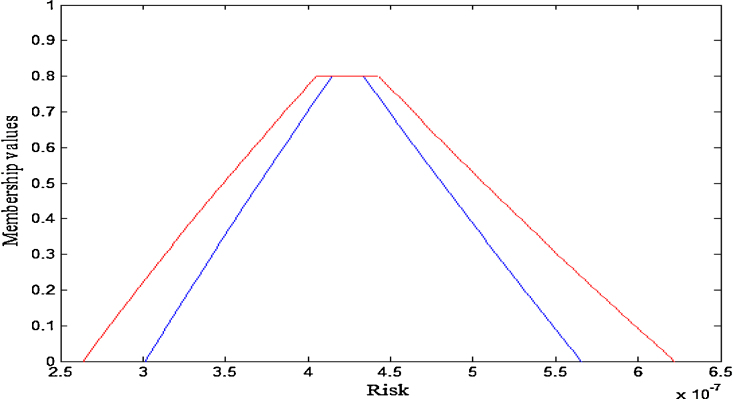
Membership function of non cancer risk at 95th fractile.

For instance, at 95th fractile, non-cancer risk value lies in the completely generalized interval valued fuzzy number whose upper membership function is [2.639e-07, 4.052e-07,4.429e-07, 6.22e-07;0.8] and lower membership function is [3.016e-07, 4.147e-07, 4.335e-07,5.655e-07; 0.8] and which is depicted in Fig. 4.

Similarly, at 85th fractile, risk values lie in the generalized fuzzy number {[2.515e-07, 3.862e-07, 4.222e-07, 5.928e-07; 0.8] (UMF); [2.874e-07, 3.952e-07, 4.132e-07 4.148e-07, 5.39e-07; 0.8] (LMF)}.

**Fig. 5:**
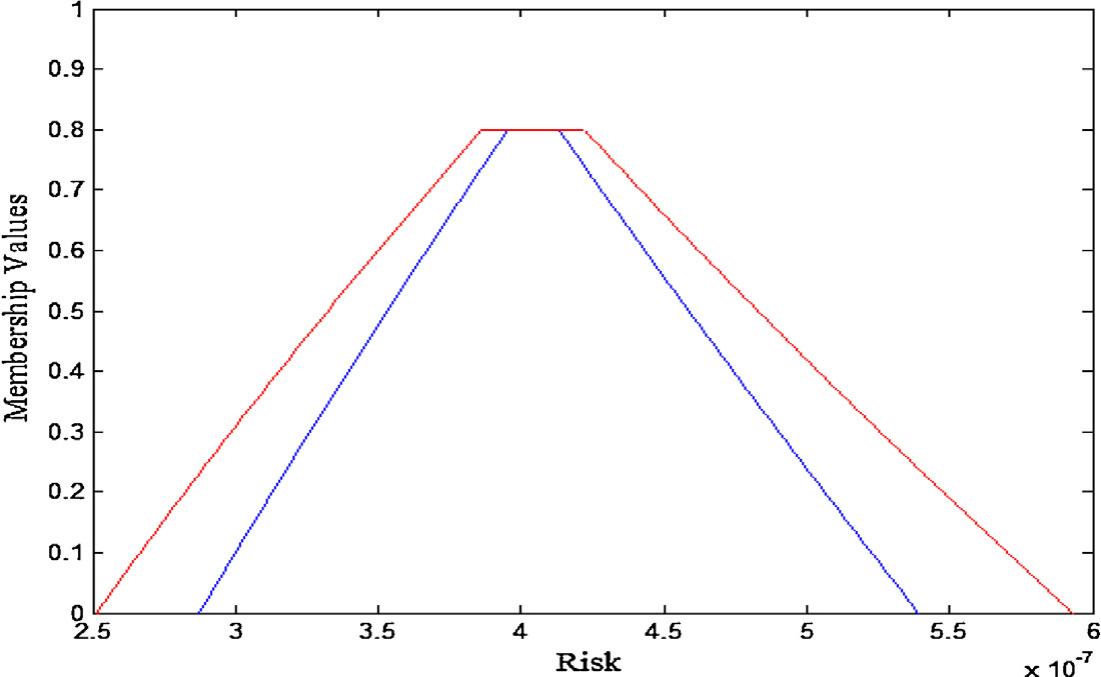
Membership function of non cancer risk at 85th fractile.

The graphical representation of the resulting non cancer risk value at 85th fractiles is depicted below in Fig. 5.

## Additional information

Health risk assessment on exposure to substance or activity subject to uncertainty has to be dealt with robust methodology in order to make optimum decision. Decision maker in health risk assessment has the responsibility to estimate the severity and likelihood of harm to humans’ health from exposure to a substance which is under plausible circumstances can cause harm. Mathematical models are often used in health risk assessment and are associated with a varying degree of uncertainty, both in the choice of model and in parameters. These models are function of many variables which are subject to uncertainty due to lack of measurement point and over-calibration, inaccurate expert judgment and subjective interpretation of available data or information [5].

Generally, uncertainty is broadly categorized into aleatory and epistemic uncertainties. Aleatory uncertainty (or simply variability) arises due to inherent variability, natural stochasticity, environmental or structural variation across space or through time, manufacturing or genetic heterogeneity among components or individuals, and variety of other sources of randomness and can be handled by traditional probability theory. On the other hand, Model epistemic uncertainty (or simply called uncertainty) arises due to incompleteness of knowledge about the world. Sources of epistemic uncertainty include measurement uncertainty, small sample size, detection limits and data censoring, ignorance about the details of the physical mechanisms and processes involved and other imperfection in scientific understanding.

In health risk assessment both variability and uncertainties co-exist. Thus, there is a need to develop special techniques, which can handle hybrid uncertainties (i.e. fuzzy and random), for carrying out risk assessment. To address this issue different effort have been made by various researchers for joint propagation of variability and uncertainty in the same computation of risk viz., Flage et al. [6], discussed probabilistic and Possibilistic treatment of epistemic uncertainties, Dutta and Ali [7] studied fuzzy focal elements in Dempster-Shafer theory of evidence: case study in risk analysis, Ali et al. [8] discussed modeling uncertainty in risk assessment using Double Monte Carlo method, Dutta and Ali [9] proposed a hybrid method to deal with aleatory and epistemic uncertainty in risk assessment, Pedroni et al. [10], [11] studied propagation of aleatory and epistemic uncertainties, Arunraj et al. [12] proposed an integrated approach with fuzzy set theory and Monte Carlo simulation for uncertainty modeling in risk assessment, Pastoor et al. [13] studied roadmap for human health risk assessment in 21st century, Farako et al. [24], [25] studied risk assessment for Salmonella in tree nuts, Salmonella in low-water activity foods and Salmonella in low-moisture foods, Zwietering [14] studied uncertainty modeling for risk assessment and risk management for safe foods, Rębiasz et al. [15] studied joint Treatment of Imprecision and Randomness in the Appraisal of the Effectiveness and Risk of Investment Projects, Alyami et al. [16] studied advanced uncertainty modeling for container port risk analysis, studied uncertainty handling in safety instrumented systems according to IEC and new proposal based on coupling Monte Carlo analysis and fuzzy sets, Abdo and Flaus [17] proposed a new approach with randomness and fuzzy theory for uncertainty quantification in dynamic system risk assessment, Zhang et al. [18] discussed risk assessment of shallow groundwater contamination under irrigation and fertilization conditions. However, in all their efforts it is observed that representation of epistemic uncertainty is of Type-I fuzzy set. But, in some situation it is not always possible for a membership function of the type *μ* : *X* → [0, 1] to precisely assign one point from [0,1] so it is more realistic to assign interval value. According to Gehrke et al. [19] many people believe that assigning an exact number to expert’s opinion is too restrictive and the assignment of an interval valued is more realistic. In such situations interval valued fuzzy set (IVFS) comes into picture. IVFS was developed in the 1970′s. In May 1975 Sambuc [20] presented in his doctoral research (thesis) the concept of IVFS named as *φ*−fuzzy set. After development of IVFVs, different researchers have been studied this issue and applied in different areas. An IVFS is a set in which every element has degree of membership in the form of an interval. One can say, IVFS consist of two membership function, one is upper membership function (UMF) and other is lower membership function (LMF). Dutta [21] presented a hybrid approach and combined probability distributions, type-I fuzzy set (normal fuzzy numbers) and generalized fuzzy numbers, Dutta [22] also presented an approach to combine probability distributions, normal fuzzy numbers and generalized interval-valued fuzzy numbers and a hypothetical case study has been carried out using the proposed approach. Dutta [23], [5] also presented approaches to deal with hybrid situations. However, all the approaches are inappropriate when representation of model parameters are probability distributions, generalized fuzzy numbers and normal interval valued fuzzy numbers (IVFNs). Therefore, it motivates us to devise a new technique to deal with such situation.

In this regard, this paper presents an approach to combine probability distributions, generalized fuzzy numbers and normal interval valued fuzzy numbers (IVFNs) within the same framework and also a case study in non-cancer risk assessment has been carried out in this setting.

In this present paper we have proposed a method to deal with such situation where some possibilistic distributions are considered as normal interval valued fuzzy numbers together with generalized fuzzy numbers. To demonstrate and make use of the proposed approach a hypothetical case study for non-cancer risk assessment is presented here. After performing risk assessment using our approach risk is obtained in the form of Cdfs and from which, risk has been evaluated in two forms. One p-box and seconds is membership functions of the risk are generated at different fractiles. The membership functions of risk at different fractiles are completely generalized interval valued fuzzy number since representation of at least one parameter is taken as generalized fuzzy number (IVFN). The upper and lower membership functions of the completely generalized interval valued fuzzy number is trapezoidal type generalized fuzzy number, because any arithmetic operation of generalized fuzzy numbers (also generalized fuzzy number and normal fuzzy number) produces trapezoidal type generalized fuzzy number.

The main disadvantage of the proposed approach is that if representation of any model parameter is generalized IVFN then it is inappropriate to deal with such model or situation.

## Conflict of interest

The authors declare that there are no conflicts of interest.

## Figures and Tables

**Table 1:** Parameters used in the risk assessment.

Parameter	Units	Type of Variable	Value/distribution
Average Time (AT)	Days	Constant	25550
Body Weight (BW)	Kg	Probabilistic	Normal (70,5)
Exposure Duration (ED)	Years	Constant	30
Exposure frequency (EF)	Days/year	Constant	350
Fraction of contaminated Fish (FR)	–	Constant	0.5
Fish Ingestion Rate (FIR)	g/day	Probabilistic	170
Conversion Factor (CF)	–	Constant	1E-09
PEC for As	ug/l	Fuzzy	[4,5, 6; 0.8]
BCF for As	l/kg	Fuzzy	[35,45,55] UMF [40,45,50] LMF
Oral Rfd for As	mg/(kg day)	Constant	3.0E–04
